# Patterns and Outcomes Associated with Patient Migration for Liver Transplantation in the United States

**DOI:** 10.1371/journal.pone.0140295

**Published:** 2015-10-15

**Authors:** Kristopher P. Croome, David D. Lee, Justin M. Burns, Dana K. Perry, Andrew P. Keaveny, C. Burcin Taner

**Affiliations:** Mayo Clinic Collaborative in Transplant Research and Outcomes, Department of Transplant, Mayo Clinic, Jacksonville Florida; ISMETT-UPMC Italy/ University of Catania, ITALY

## Abstract

**Background:**

Traveling to seek specialized care such as liver transplantation (LT) is a reality in the United States. Patient migration has been attributed to organ availability. The aims of this study were to delineate patterns of patient migration and outcomes after LT.

**Study Design:**

All deceased donor LT between 2008–2013 were extracted from UNOS data. Migrated patients were defined as those patients who underwent LT at a center in a different UNOS region from the region in which they resided and traveled a distance > 100 miles.

**Results:**

Migrated patients comprised 8.2% of 28,700 LT performed. Efflux and influx of patients were observed in all 11 UNOS regions. Regions 1, 5, 6, and 9 had a net efflux, while regions 2, 3, 4, 7, 10, and 11 had a net influx of patients. After multivariate adjustment for donor and recipient factors, graft (p = 0.68) and patient survival (p = 0.52) were similar between migrated and non-migrated patients.

**Conclusion:**

A significant number of patients migrated in patterns that could not be explained alone by regional variations in MELD score and wait time. Migration may be a complex interplay of factors including referral patterns, specialized services at centers of excellence and patient preference.

## Introduction

Many patients in the United States pursue health care treatment outside of their immediate geographic area in a concept coined “destination health care^”[^[1,2]. Patients’ reasoning for travel can vary and may include: physician referral patterns, insurance contracting, availability of specialized services at centers of excellence or simply patient preference. The phenomenon of patient migration has recently been brought into the spotlight in the field of transplantation[3–5]. Candidates for solid organ transplantation can be listed at any transplant center in the country, provided that they can reach that center at the time of organ offer. Liver transplantation (LT) rules were modified in 2002 such that liver graft allocation is preferentially offered in a stepwise fashion to the candidate with the highest Model for End-Stage Liver Disease (MELD) score within a local donation service area (DSA), followed by allocation within the same United Network for Organ Sharing (UNOS) region and then nationally (outside of a given UNOS region). Regional variations in MELD score at LT, wait time and waitlist mortality rates have contributed to patients, migrating to UNOS regions with shorter wait time, in order to receive LT more quickly^4^. To date, very few studies examining LT candidate migration have been published[3–6]. In addition, the definition of the “migrated patient” has been inconsistent: a single center experience defined a “migrated patient” as one who was listed at one center and transplanted elsewhere; another study investigated patients who were dually listed using national data[3,4]. To date, no consensus definition for the migrated patient exists and no national studies have investigated, in detail, the patterns of LT candidates’ migration from one UNOS region to another. The present study aimed to describe the number and patterns of patient migration and what effects, if any, these migrations had on graft and patient survival compared to those who pursued LT in their UNOS region of residence using national data over a 5-year time period.

## Materials and Methods

After approval from the Mayo Clinic Institutional Review Board, data were obtained and extracted from the UNOS Standard Analysis and Research file. None of the transplant donors were from a vulnerable population. All patient records and data was anonymized and de-identified prior to analysis. The study population included all deceased donor LT recipients performed in the US from June 17, 2008 to June 16, 2013. This period was chosen to encompass the 5-year time period prior to the inception of Share 35 rule, which preferentially allocated liver grafts at a regional level to patients with a MELD score of ≥35. Pediatric (age<18years), and multi-organ transplants (except simultaneous liver-kidney transplants) were excluded from this analysis. Non-US residents were also excluded from the analysis.

Patients’ home region of residence and UNOS region in which they received LT were extracted using US Postal zip codes. Distances between a patients home address and transplanting centers was calculated using Google’s mapping API software. Migrated patients were defined as those patients who underwent LT at a transplant center that was in a different UNOS region from the region in which they reside and traveled greater than 100 miles. One hundred miles was chosen in order to exclude patients who were travelling to transplant centers that were in close proximity but located in a different UNOS region. A cutoff of 100 miles has previously been shown to be associated with worse survival in a study of US veterans undergoing LT[7]. Graft survival was calculated from the time of LT until death, graft loss or date of last follow-up. The occurrence and the date of death were obtained from data reported to the SRTR by the transplant centers and were completed by data from the US Social Security Administration and from the OPTN. Potentially confounding donor and recipient factors were examined including the donor risk index (DRI)[8], recipient age, MELD score at transplant, body mass index (BMI), gender, race, insurance status, retransplant status, presence of hepatocellular cancer (HCC) as secondary diagnosis, MELD exception points for HCC, and liver disease etiology. Multiple listing was defined as those patients listed at different transplant centers during the same time interval.

All statistical analyses were performed using STATA 12 (Stata Corp., College Station, TX). Differences between groups were analyzed using the unpaired *t* test for continuous variables and by the χ^2^ test or continuity correction method for categorical variables. Wilcoxon rank-sum was used for variables that did not display a normal distribution. Survival curves for patient or recurrence free survival were generated using the Kaplan-Meier method and compared by the log-rank test. All statistical tests were two-sided and differences were considered significant when p < 0.05.

## Results

**Fig 1:**
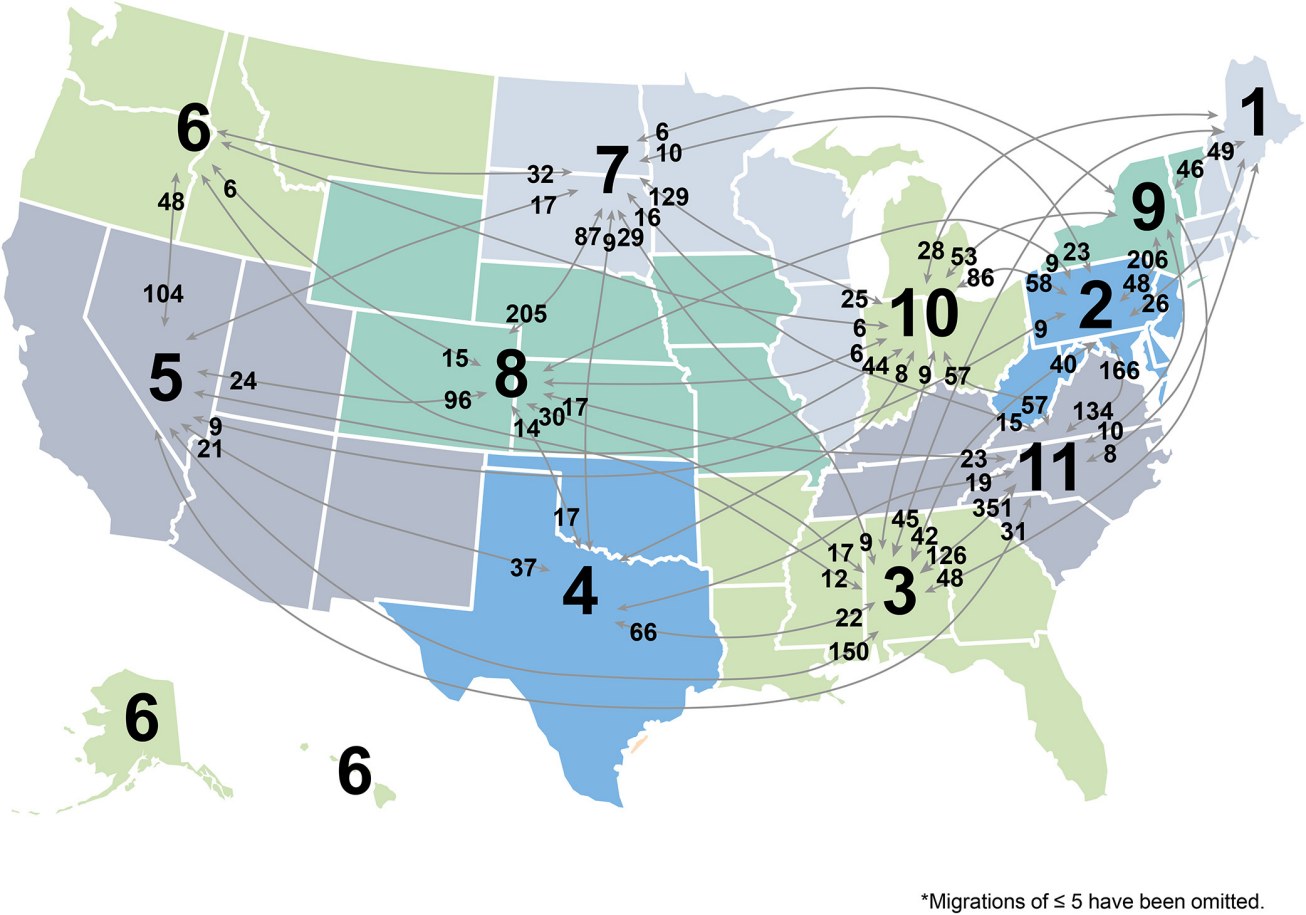
Detailed summary of recipients' home UNOS regions and the UNOS regions they received LT.

A total of 28,700 LT were performed in the study time period. A breakdown of the UNOS regions where each of these patients initially resided and where they were ultimately transplanted can be seen in **Fig 1, Table 1**. This included 26,345 (91.8%) patients who were transplanted in their local UNOS Region and 2355 (8.2%) patients who migrated to another UNOS Region and traveled greater than 100 miles for LT. There was no significant difference in the number of patients who migrated by year from 2008–2013 (p = 0.41). Mean travel distance from the patients’ home to their transplant center was 740.9 ± 696.8 miles (median: 478.4 miles) for patients who migrated and was 71.9 ± 149.0 (median: 31.6 miles) for those who did not migrate (p<0.001). There was no difference in recipient age between migrated (mean: 54.7 ± 10.5 years) and non-migrated patients (mean: 54.4 ± 10.3 years) (p = 0.15). Migrated patients were more likely to be male (72.5% vs. 66.4%; p<0.001), were more likely to be identified ethnically as white (78.3% vs. 70.3%; p<0.001) and had a lower BMI (27.7 ± 5.5 vs. 28.4 ± 5.7; p<0.001). Migrated patients were less likely to have positive serology for hepatitis C virus (38.1% vs. 42.8%; p<0.001), were less likely to have alcohol related cirrhosis (7.8% vs. 11.3%; p<0.001) and were less likely to have received MELD exception points for HCC (21.1% vs. 23.9%; p = 0.002). Migrated patients were more likely to have a cholestatic etiology of their liver disease (12.4% vs. 7.5%; p<0.001). A significantly higher proportion of migrated patients were candidates for re-transplants (7.3% vs. 6.2%; p = 0.04). A smaller proportion of patients with blood group B and AB migrated compared to those that did not migrate (11.6% vs. 13.8%; p = 0.003 and 3.9% vs. 4.9%; p = 0.04, respectively). The proportion of patients with blood groups O and A who migrated and non-migrated was not significantly different (46.2% vs 44.7%; p = 0.17 and 38.2% vs. 36.5%; p = 0.11, respectively). The proportion of patients listed at multiple transplant centers was more common in patients who migrated compared to those who did not migrate (25.0% vs. 5.4%; p<0.001).

**Table 1:** Detailed summary of recipients' home UNOS regions and the UNOS regions they received LT.

					Transplanting Region							
Home Region	1	2	3	4	5	6	7	8	9	10	11	
**1**	**1125**	26	45	0	1	0	4	4	46	28	8	1287
**2**	3	**2916**	42	1	9	0	10	4	206	86	134	3411
**3**	4	40	**4391**	66	4	0	29	30	3	9	351	4927
**4**	1	9	22	**2295**	21	2	9	14	1	8	19	2401
**5**	0	5	150	37	**3831**	48	17	96	4	44	31	4263
**6**	0	3	12	1	104	**665**	32	15	0	6	4	842
**7**	1	23	9	1	0	1	**2258**	205	0	25	15	2538
**8**	0	9	17	17	24	6	87	**1697**	1	6	23	1887
**9**	49	48	48	1	1	0	6	1	**1448**	53	10	1665
**10**	0	58	3	1	1	0	129	3	1	**2175**	57	2428
**11**	1	166	126	4	1	0	16	17	4	57	**2494**	2886
**Missing**	1	16	65	12	12	1	26	5	2	22	3	165
	1185	3319	4930	2436	4009	723	2623	2091	1716	2519	3149	28700

Time on the waitlist at the transplanting center was significantly shorter for migrated patients compared to those who did not migrate (221.5 ± 430.7 days vs. 266.1 ± 522.9 days, respectively; p<0.001). There were no significant differences in donor age between migrated and non-migrated LT recipients (40.4 ± 16.7 versus 41.1 ± 16.5, p = 0.05), or DRI of the graft (1.40 ± 0.35 versus 1.41 ± 0.35, p = 0.1). Biological MELD score at the time of LT was lower for migrated patients versus those who did not migrate (21.6 ± 10.1 vs. 22.3 ± 10.7, p<0.001), match MELD score was also lower for migrated patients in comparison to those who did not migrate (25.6 ± 7.1 vs. 26.8 ± 10.2, p<0.001). Recipient characteristics for the two study groups can be seen in **Table 2**.

**Table 2:** Recipient characteristics in migrated and non-migrated patients.

	Migrated	Non-Migrated	
Recipient Characteristics	N = 2355	N = 26345	p value
**Age at transplant (years)**	54.7±10.5	54.4±10.3	0.15
**Body mass index (kg/m** ^**2**^ **)**	27.7±5.5	28.4±5.7	<0.001
**Gender (male)**	1708 (72.5%)	17484 (66.4%)	<0.001
**Diagnosis**			
HCV	896 (38.1%)	11279 (42.8%)	<0.001
EtOH	183 (7.8%)	2989 (11.3%)	<0.001
NASH	162 (6.9%)	1935 (7.3%)	0.41
Cholestatic	291 (12.4%)	1972 (7.5%)	<0.001
**MELD exception score for HCC**	496 (21.1%)	6283 (23.9%)	0.002
**Calculated MELD score**	21.6±10.1	22.3±10.7	<0.001
**Match MELD score**	25.6±7.1	26.8±10.2	<0.001
**Re-transplant**	173 (7.3%)	1645 (6.2%)	0.04
**Distance traveled (miles)**	740.9±696.8	71.9±149.0	<0.001
**Multi-listed**	589 (25.0%)	1419 (5.4%)	<0.001
**Days on waitlist (at transplanting center)**	221.5±430.7	266.1±522.9	<0.001
**Race/ethnicity**			
White	1843 (78.3%)	18517 (70.3%)	<0.001
Black	148 (6.3%)	2738 (10.4%)	<0.001
other	364 (15.5%)	5090 (19.3%)	<0.001
**Private insurance**	1433(60.9%)	15113 (57.4%)	<0.001
**ABO Blood group**			
**O**	1088 (46.2%)	11785 (44.7%)	0.17
**A**	900 (38.2%)	9628 (36.5%)	0.11
**B**	274 (11.6%)	3641 (13.8%)	0.003
**AB**	93 (3.9%)	1291 (4.9%)	0.04

EtOH = Alcoholic Cirrhosis; DBD = Donation after brain death; DCD = Donation after cardiac death; HBV = Hepatitis B virus; HCV = Hepatitis C virus; NASH = Non-alcoholic steatohepatitis; HCC = Hepatocellular carcinoma

A summary of the number of patients migrating in and out of each region can be seen in **Table 3**. UNOS Region 3 had the highest total number of LT performed (n = 4930) while UNOS Region 6 had the least (n = 723). Regions who had a net efflux of patients included: Region 5 (n = -266), Region 9 (-178), Region 6 (n = -119) and Region 1 (n = -68). Regions who had a net absolute influx of patients included: Region 11 (n = 197), Region 10 (n = 122), Region 7 (n = 109), Region 8 (n = 92), Region 3 (n = 66), Region 2 (24) and Region 4 (n = 22). In a separate subanalysis, when transplant centers were divided into quartiles based on transplant volume and high volume centers were defined as those above the 75^th^ percentile for transplant volume, the majority of migrated patients traveled to the high volume centers (n = 1790; 76%).

**Table 3:** Total number of recipients originating from each UNOS region (Pts with Perm Address), number of LT performed in each region (Total LT Performed) and migration in and out of each region.

Region	Pts with Perm address in Region	Total LT Performed	Emigrated out of Region	Immigrated to Region	Net Migration
**1**	1287	1185	120	52	-68
**2**	3411	3319	219	243	24
**3**	4927	4930	399	465	66
**4**	2401	2436	106	128	22
**5**	4263	4009	428	162	-266
**6**	842	723	176	57	-119
**7**	2538	2623	151	260	109
**8**	1887	2091	170	262	92
**9**	1665	1716	201	23	-178
**10**	2428	2519	144	266	122
**11**	2886	3149	240	437	197
**Unknown**	165	0	NA	NA	NA

Pts = Patients; LT = Liver Transplantation

A total of 2008 (7.0%) patients were multi-listed at more than one transplant center during the study period. The proportion of multi-listed patients transplanted within each respective region was highest in UNOS Region 4 (22.9%) and lowest in UNOS Region 5 (3.7%). A subgroup analysis was performed looking only at those patients who migrated to another UNOS Region and were multi-listed: a total of 589 patients, who migrated and were multi-listed, were identified. This group accounted for 2.1% of the total number of patients transplanted in the study time period. When looking at the UNOS region from which patients migrated, the proportion of patients who migrated and were multi-listed was: Region 9 (5.3%), Region 5 (5.0%), Region 1 (4.9%), Region 6 (1.9%), Region 2 (1.6%), Region 8 (1.6%), Region 7 (1.1%), Region 4 (1%), Region 3 (1%), Region 11 (0.66%) and Region 10 (0.5%). The same cohort of patients migrated to and received their transplant in the following UNOS regions: Region 10 (4.5%), Region 3 (4.5%), Region 11 (2.7%), Region 8 (2.1%), Region 4 (1.9%), Region 6 (1.0%), Region 7 (1.0%), Region 2 (0.7%), Region 1 (0.3%), Region 5 (0.3%) and Region 9 (0.1%).

**Fig 2:**
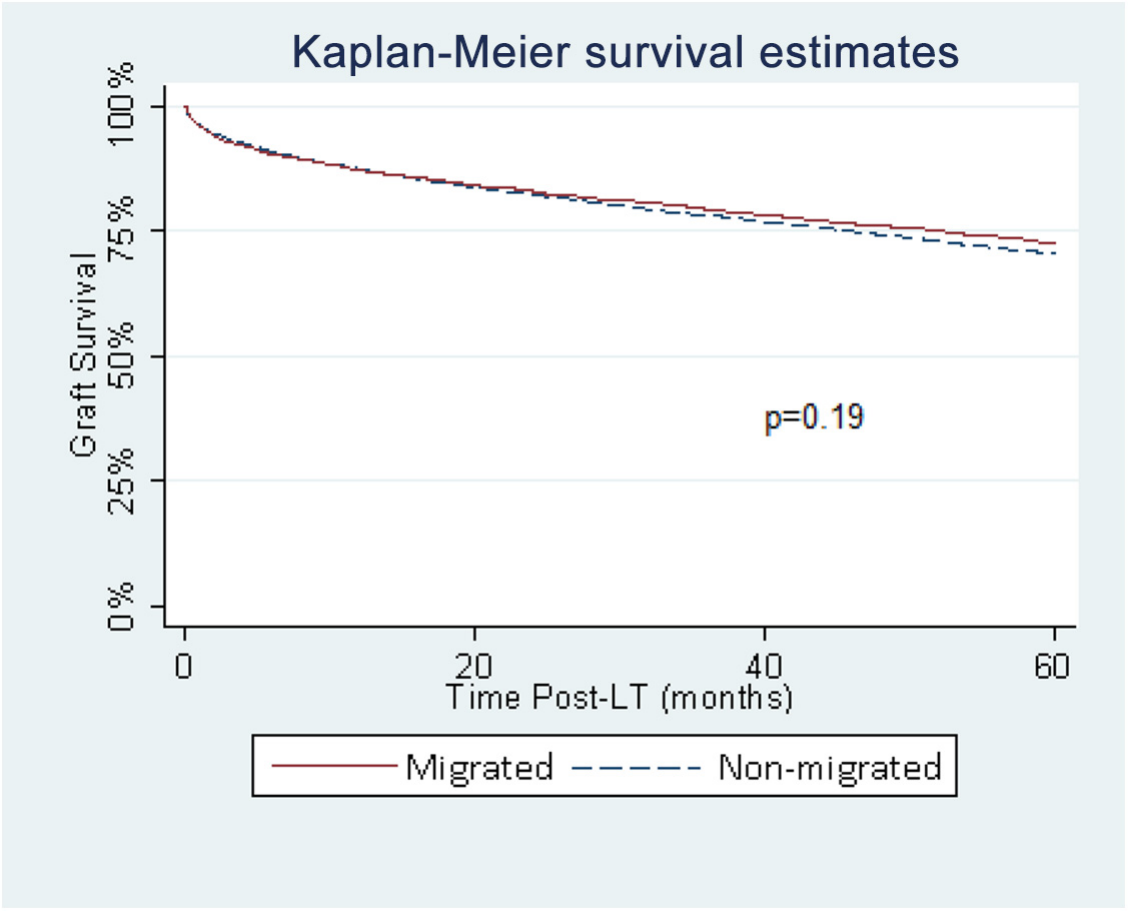
Graft survival in migrated and non-migrated groups.

**Fig 3:**
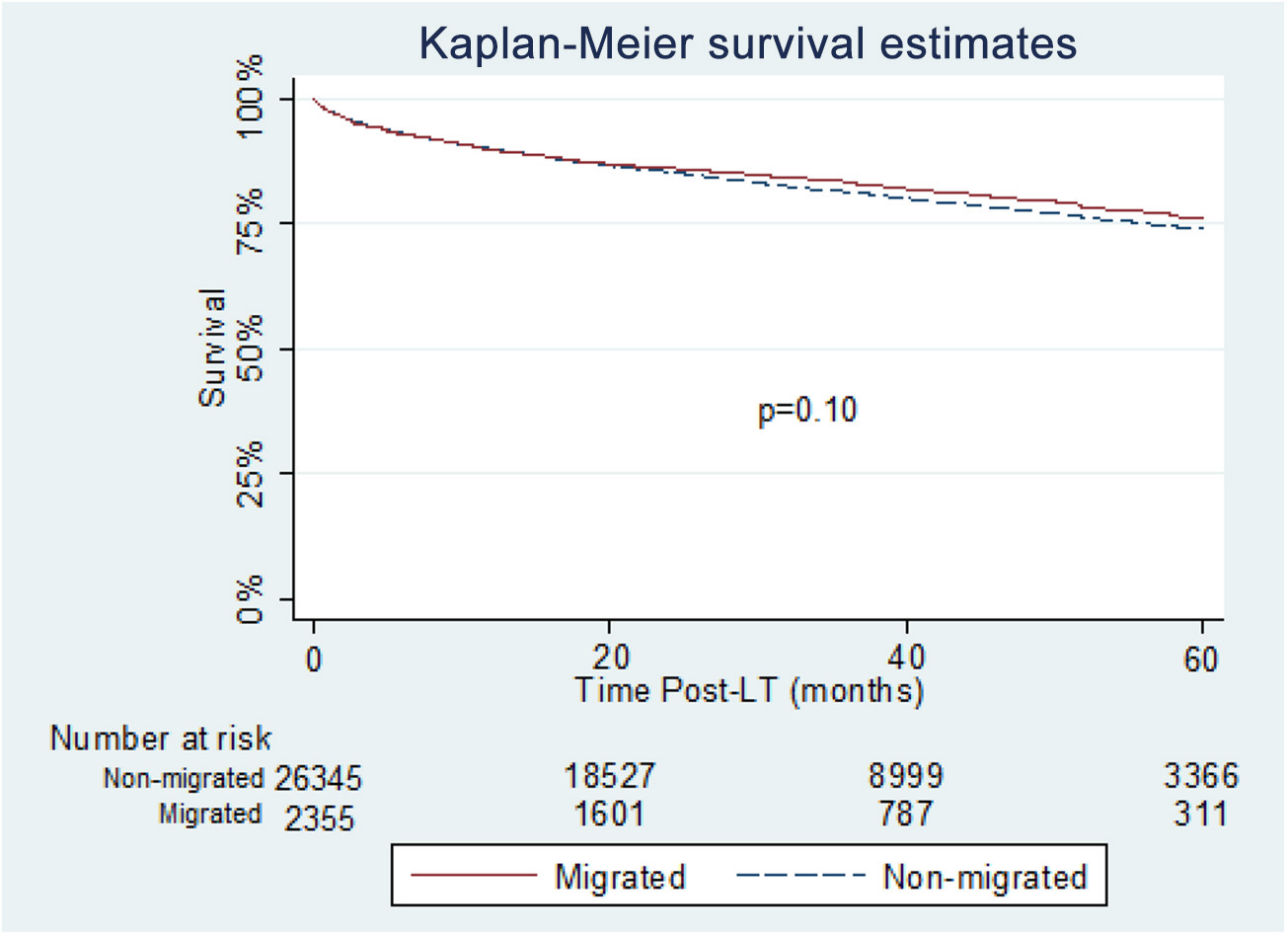
Patient survival in migrated and non-migrated groups.

On unadjusted analysis, there was no significant difference in graft or patient survival between migrated and non-migrated patients (p = 0.19 and p = 0.10, respectively) (**Fig 2 and Fig 3)**. A multivariate Cox proportional Hazards model, adjusting for both donor and recipient factors, showed migrating to another region (p = 0.68) or multi-listing (p = 0.24) were not associated with graft survival. A separate multivariate Cox proportional Hazards model looking at patient survival showed migrating to another region or multi- listing were also not significant (p = 0.52) and (p = 0.06).

## Discussion

The current study describes patterns of patient migration between UNOS Regions for LT between June 2008 and June 2013. Migration was shown to involve efflux and influx of patients in all 11 UNOS Regions in the 5 year era before implementation of the Share 35 rule. In addition, we demonstrated similar graft and patient survival between patients who migrated versus non-migrated.

An important challenge in LT practice today is the disparity between the need for and availability of donor liver grafts as the number of patients added to the national waiting list continues to increase[9]. Regional variations in MELD score at LT and waitlist mortality are thought to be downstream effects of differences in liver graft availability and use[10–12]. We found in the study period that a significant proportion of patients (8.2%) migrated out of their region of residence in order to be transplanted. Due the limitations of the SRTR database, it is not possible to provide specific data on the exact reason(s) for migration. However, the fact that all 11 UNOS regions had both immigration and emigration suggests that migration is a complex process with many underlying factors. Indeed, a large number of patients migrated to regions typically regarded as having longer wait times (Regions 2, 5, and 7). Without question, a subset of patients fit the perceived stereotypical definition of the “migrated patient” (traveling to a region with shorter waiting times); however, this does not represent all of migrated patients.

The data presented in this report shows slightly lower biologic MELD and LT match MELD scores in migrated compared to non-migrated patients. As has been demonstrated in previous studies, migrated patients were more likely to be male, of white race, to have liver disease of cholestatic etiology and to have private insurance[4,5]. Of significant interest was the finding that a lower proportion of patients with MELD exception points for HCC were seen in the migrated compared to non-migrated group. This pattern of migration may expose a flaw in the current allocation system, whereby patients who are accruing additional MELD exception points (such as patients with HCC) do not feel compelled to migrate while patients with cholestatic liver disease may have a stagnant biological MELD score while experiencing significant complications from their disease with possible risk for mortality[13–15].

In multivariate analysis, we did not find differences in post-transplant patient or graft survival between the migrated and non-migrated groups. This is in contrast to a recent single center study that showed an inferior 5 year patient survival in migrated LT candidates[5]. It is possible that this difference could be explained by the small sample size or selection bias in the latter study. Assuming that migrated patients return to their home region following LT episode, these patients had similar long-term survival compared to patients that stayed in their local region for LT. National consensus and practice regarding the appropriate long-term management of LT recipients may explain the similar outcomes in this contemporary patient population[16].

Since the present study only examined patients who received LT, we cannot comment on what effect shorter wait times could have had on waitlist mortality or the cost for patients who migrated^7^. It has previously been shown that a longer wait time and subsequent LT at higher MELD score resulted in higher cost secondary to reasons such as repeat hospitalization and increased morbidity before and after LT[17–19]. It is also uncertain what consequences patient migration to another region had on the region from which they migrated. It is conceivable that this may indeed have shortened the regional wait time for those patients who did not migrate, due to greater availability of organs for those individuals. An unintended consequence of patient migration might be a move towards equilibrium in wait times between the UNOS regions. Since the analysis here is focused on only patients who underwent LT, we avoid speculation regarding the impact on wait times and waitlist mortality.

In addition to migration, patients are permitted to be evaluated and listed at more than one transplant center simultaneously, a concept referred to as multi-listing. It has previously been suggested that candidates located in “competitive DSAs” may undergo multi-listing in a second DSA that allocates liver grafts at lower MELD scores with shorter waiting times[3]. Multi-listed candidates were studied previously in the pre-MELD allocation era by Merion et al. In their study, 3.3% of LT candidates were multi-listed and post-transplant mortality was similar between the multi-listed and single-center listed recipients. In the present study, multi-listed patients represented 25% of our defined migrated patients. Using only the multi-listed patient to define the “migrated patient” will grossly underestimate the number of patients who truly migrate.

The current study provides an important perspective into patient migration for LT in the US. While it is clear that geographic differences in organ availability and waitlist time are a national issue, it is unclear if revamping the UNOS regions will decrease patient migration. Our healthcare system values patient autonomy in choosing where to seek care and does not restrict patient migration for transplant or any other illness. The large proportion of migrated patients that sought care at high volume centers may reflect case complexity and recognized specialized care at centers of excellence. Fitting with this notion, a higher number of re-transplants were seen in the migrated group. It is possible that these patients were rejected at smaller local centers and migrated to a larger program in another UNOS region more willing to tackle the burden of a higher risk case[20]. Undoubtedly, there is an underlying cost to all travel. However, as we move forward with discussions on redistricting, it will be important to consider the spectrum of financial issues related to any change. Cost projections of LT at high MELD scores, related care of a sicker patient, cost-effectiveness at high volume centers, procurement team travel versus cost of patient travel and accommodation should be investigated in future studies.

The proposed definition of the “migrated patient” in this study is a reasonable attempt to accurately capture those patients that travelled a distance to another UNOS region in order to receive LT and therefore can be more broadly adopted. Limitations of our study include its reliance on registry data as well as lack of data on patients’ reasoning for migration and the impact of patients’ travelling on a given center’s wait list. Future studies could investigate the driving force(s) that cause patients to seek LT at centers that are not in their general vicinity. We limited our analysis to the 5 years prior to implementation of the Share 35 rule in order to avoid differences in patient migration that may have arisen as a consequence of this policy. Subsequent studies could compare patient migration in the pre- and post-Share 35 eras.

In conclusion, the present study provides a detailed description of patient migration and fills a gap in the overall picture of LT practice in the US. Regional variation in MELD score at LT is undoubtedly a driving force for a subset of patients to migrate. However, this study demonstrated that patient migration for LT may be a complex interplay of many factors such as physician referral patterns, insurance contracting, availability of specialized services at centers of excellence and patient preference. In addition, patient migration did not result in any difference in patient or graft survival following LT.

## Abbreviations

CI: Confidence interval
CIT: Cold ischemia time
CT: Computed tomography
DCD: Donation after Cardiac Death
DBD: Donation after Brain Death
DRI: Donor risk index
HCC: Hepatocellular carcinoma
LT: Liver transplant
MELD: Model for end stage liver disease
NASH: Non-alcoholic steatohepatitis
SD: Standard deviation
UNOS: United Network for Organ Sharing
